# Bladder malignancy as a cause of spontaneous bladder rupture: A systematic review

**DOI:** 10.1002/bco2.281

**Published:** 2023-08-30

**Authors:** Johnson Da Huang, Emily Ximin Shao, Chui Ming Tham, Eric Chung, Handoo Rhee

**Affiliations:** ^1^ University of Queensland Brisbane Queensland Australia; ^2^ Department of Urology, Princess Alexandra Hospital University of Queensland Woolloongabba Queensland Australia

**Keywords:** bladder cancer, spontaneous rupture, systematic review, urothelial cell carcinoma (UCC)

## Abstract

**Objectives:**

To characterise cases of spontaneous rupture of the urinary bladder in the context of bladder cancer.

**Methods:**

A systematic review was performed to characterise cases of spontaneous bladder rupture in patients with bladder cancer. The Preferred Reporting Items for Systematic Reviews and Meta‐Analyses (PRISMA) system was utilised, with databases being searched for relevant cases. Patient characteristics were extracted, including age, sex, presenting signs and symptoms, management modalities, tumour histology and mortality.

**Results:**

Thirty cases were included. Seventeen (57%) were male, and the median age of presentation was 59. Abdominal pain and peritonism were the most common presenting symptoms, in 80% and 60% of patients, respectively. Most patients (*n* = 16, 53%) had urothelial cell carcinoma. Nine patients (30%) died during their initial hospitalisation.

**Conclusion:**

Spontaneous bladder perforation in the context of bladder cancer is a rare cause of acute abdomen. The diagnosis is associated with high mortality, highlighting the aggressive nature of the malignancies that cause spontaneous bladder rupture. This raises important questions about the role of emergency cystectomy, the timing of systemic therapy and the appropriate involvement of palliative care.

## 1 INTRODUCTION

Spontaneous rupture of the urinary bladder occurs when there is a rupture or perforation of the bladder wall without trauma or instrumentation. It is a rare surgical emergency with significant morbidity and mortality.^1^ Diagnosis of this condition can be delayed due to the obscure presenting signs and symptoms, ambiguous imaging finds and rarity of the event.^1^

More common causes of spontaneous rupture of the urinary bladder include bladder outlet obstruction, overdistention, inflammation and infection.^2^ Weakening of the bladder wall and neurogenic processes that reduce the awareness of bladder distension contribute to the pathogenesis of spontaneous bladder rupture, with diabetes, alcohol intoxication and post‐radiotherapy being major associations.^2^ A rare cause of spontaneous urinary bladder rupture is malignancy, often presenting as advanced disease (locally and systemically) with a poor prognosis.

There have been several case reports and case series on the spontaneous rupture of the urinary bladder secondary to malignancy; however, no systematic reviews have been published on the topic. We performed a systematic review to highlight the epidemiological characteristics of patients presenting with this condition, collate the presenting symptoms, diagnostic and treatment approaches and prognosis.

## 2 MATERIALS AND METHODS

**FIGURE 1:**
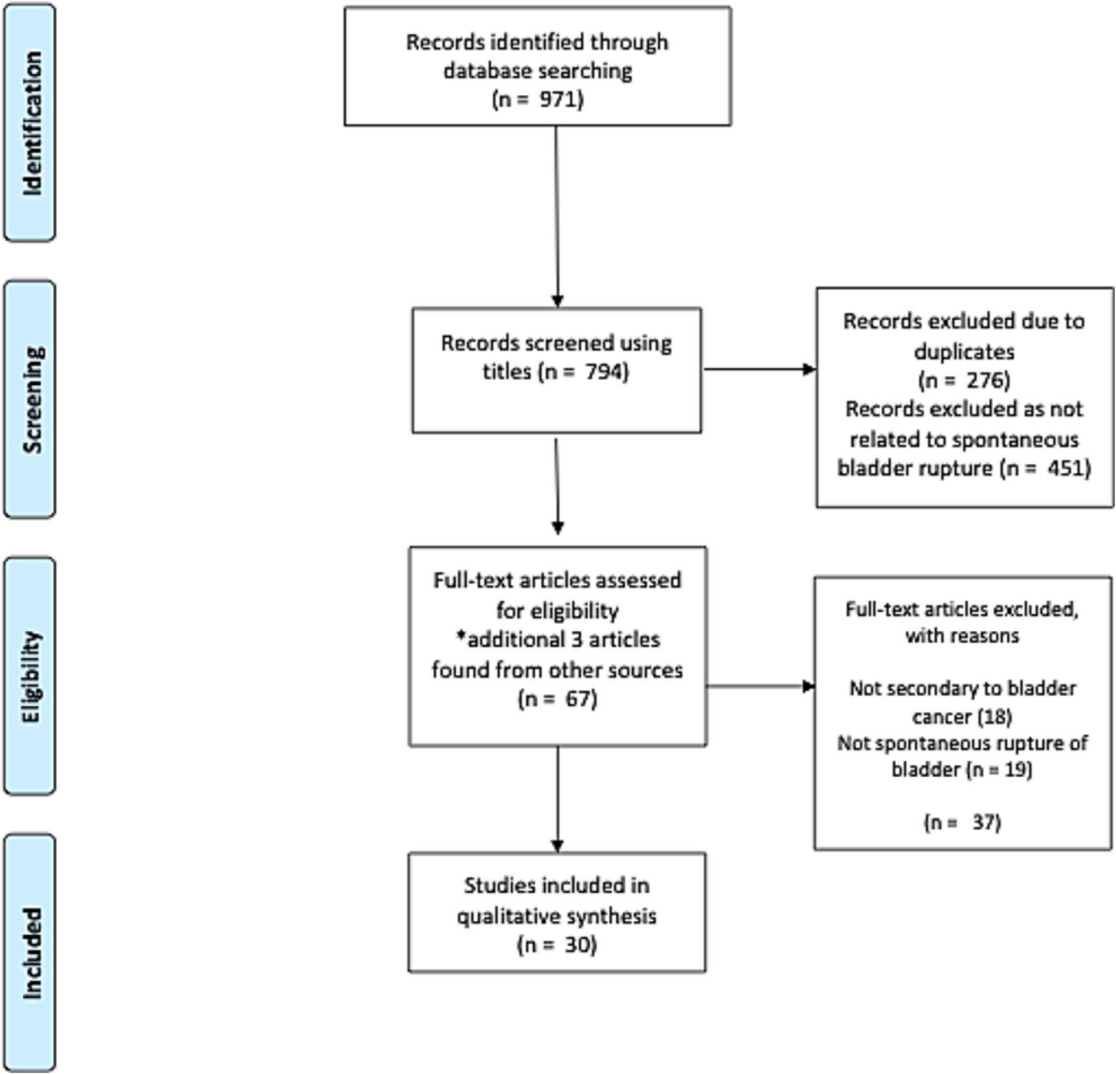
Preferred Reporting Items for Systematic Reviews and Meta‐Analyses Search methodology flow diagram—used in systematic review search using the search terms: ((urinary bladder rupture) OR (bladder rupture) OR (rupture)) AND ((urinary bladder cancer) OR (bladder cancer) OR (urinary bladder neoplasms) OR (bladder tumor) OR (bladder neoplasms)). *Three articles that were noted from review articles^1^, ^4^, ^5^ that were not identified in the PubMed and EMBASE search were included.

A systematic review of the existing literature was conducted to explore the literature on spontaneous bladder rupture in the context of bladder cancer. The Preferred Reporting Items for Systematic Reviews and Meta‐Analyses (PRISMA) system was utilised.^3^ Case reports and studies were identified from PubMed and EMBASE using the search terms noted in Figure 1. The search was performed in April 2023. All case reports or case series of patients with spontaneous urinary bladder rupture in the context of active bladder cancer were included (not postoperative). Any case where the bladder rupture occurred post‐resection was excluded. Articles that were not peer reviewed and those published in languages other than English were excluded. In addition, references of eligible review articles were also surveyed for appropriate cases. Two reviewers (J.H. and E.S.) independently screened the articles for eligibility and exclusion. Disagreements were resolved by a third reviewer (H.R.).

Additionally, the case details of a patient presenting with spontaneous rupture of the bladder presenting at our institution (Princess Alexandra Hospital, Woolloongabba, Australia) were included. Informed consent was obtained from this patient for de‐identified clinical and imaging information.

Data were extracted on patient characteristics, including patient age, sex, presenting signs and symptoms, management modalities, tumour histology and mortality. Characteristics were tallied, and descriptive statistics were calculated.

## 3 RESULTS

The search resulted in 971 records. After duplicates were removed and articles were screened, there were 30 cases included, including our case report (Figure 1). Details of the cases can be found in Table S1.

There were 17 male (57%) and 13 female (33%) cases with a median age of 59 (Table 1). Most patients were diagnosed with an acute abdomen (73%). Abdominal pain and peritonism were the most common presenting symptoms. Other common presentation features were haematuria, nausea, vomiting and abdominal distension.

**TABLE 1:** Frequencies of patient characteristics, presenting symptoms, management, and cancer histology.

Patient characteristics	
Age (median, IQR)	59 (22.5)
Sex	
Male (%)	17 (57%)
Female (%)	13 (33%)
Presenting symptoms	
Abdominal pain (%)	24 (80%)
Peritonism (%)	18 (60%)
Abnormal symptoms of urination (%)	8 (27%)
Haematuria (%)	6 (20%)
Nausea or vomiting (%)	5 (17%)
Abdominal distension (%)	4 (13%)
Constitutional symptoms (%)	2 (7%)
Clinical diagnosis of acute abdomen	
Yes (%)	22 (73%)
No (%)	5 (17%)
Unknown (%)	3 (10%)
Pre‐operative diagnosis of bladder rupture	
Yes (%)	7 (23%)
No (%)	23 (77%)
Management	
Catheterisation (%)	23 (77%)
Laparotomy (%)	23 (77%)
Cystectomy (partial or radical) (%)	8 (27%)
Nephrostomy or ureterostomy (%)	7 (23%)
Cancer histology	
Urothelial cell carcinoma	16 (53%)
Squamous cell carcinoma	10 (33%)
Unknown or other^a^	4 (13%)
Outcome	
Death during admission (%)	9 (30%)
Recovery from acute episode, death within 180 days (%)	5 (17%)
Recovery and no further detail (%)	13 (43%)
Unknown (%)	3 (10%)

^a^ Three unknown and one inflammatory myofibroblastic tumour.

Twenty‐three patients (77%) underwent laparotomy, and 23 cases (77%) noted catheterisation in the case report. Eleven patients (37%) received a CT during their initial work‐up, but only seven were suggestive of bladder perforation (Table 1).

Sixteen patients (53%) had urothelial cell carcinomas (UCCs) of the bladder, 10 (33%) had squamous cell carcinoma (SCC), three were unspecified (10%) and one had a myofibroblastic tumour. Nine patients (30%) died during their hospitalisation. Out of those who died, three were due to complications of UCC, four were due to SCC and two were unspecified. A further five patients (17%) died within 180 days of their initial presentation (Table 1).

## 4 DISCUSSION

Bladder rupture is a potentially life‐threatening event. It is usually associated with blunt or penetrating trauma, instrumentation including catheterisation and anatomical outflow obstruction resulting in a distended bladder.^6^ It can also occur from previous bladder surgery, such as cystoscopy or bladder resection. The distinction between routine bladder rupture and spontaneous bladder rupture can be vague. Spontaneous bladder rupture is generally distinguished by aetiology: It occurs without trauma, surgery or instrumentation. Numerous factors contribute to spontaneous bladder rupture. These factors include a reduction of bladder wall integrity (such as through pelvic radiation), neurogenic mechanisms that impair awareness of bladder filling and the need to void (such as diabetes mellitus, alcohol intoxication or spinal cord injury) and increased intraperitoneal pressure (such as intrapartum vaginal delivery).^2^, ^7^ In the context of bladder carcinoma, the carcinoma reduces the integrity of the muscle wall, but spontaneous bladder rupture secondary to this is extremely infrequent.^8^ It heralds delayed presentation and diagnosis, with advanced disease and poor prognosis.^8^

The epidemiological characteristics of patients included in this study differ from that of the broader population of patients diagnosed with bladder cancer. The average age of patients diagnosed with bladder cancer is 70 years old, with a male‐to‐female ratio of 4:1.^9^ In this study, the median age was much younger at 59 years old, and the male‐to‐female ratio was 1.7:1. A review of patients with spontaneous bladder rupture (although excluding bladder cancer patients) found that spontaneous bladder rupture was more common in women.^2^ They note vaginal delivery as a risk factor for this.^2^ Post‐menopause, the bladder can become thinner and lose elasticity in women; a combination of hormonal and post‐pregnancy factors may partially account for why there seems to be a proportionally higher number of women in this review of bladder rupture secondary to malignancy.

In this review, spontaneous bladder rupture was the first presentation of bladder cancer in most cases. Only two patients had treatment prior: one with radiotherapy 3 days before presentation^6^ and one treated with immunotherapy and presenting after the second treatment cycle of pembrolizumab.^10^ As mentioned, pelvic radiation is a risk factor for spontaneous bladder rupture, and this could be both in the short and long‐term. The combination of radiation cystitis with friable, malignant tissue is the likely pathology behind this in the short term.^11^ Long‐term radiation effects on the bladder include fibrosis of the bladder wall, reducing the elasticity of the tissue, which predisposes to rupture.^11^ In the case of immunotherapy, tumour regression and inflammation induced by the treatment may have overwhelmed tissue repair mechanisms, and this is likely to have contributed to perforation.^10^

The presentation of spontaneous bladder rupture is usually with abdominal pain and symptoms of peritonism.^2^ The most common presentation of bladder cancer is painless macroscopic haematuria, with urinary frequency or urgency symptoms.^12^ One fifth of patients in this review presented with haematuria, and just under one third of cases also had urinary symptoms. This raises the importance of these symptoms in predicting bladder perforation causing acute peritonitis (Table 1). The investigation of choice for suspected bladder injury in the setting of trauma is a cystogram.^13^ However, only two patients from our analyses received a CT cystogram, with most patients that were imaged receiving X‐ray films which may be inadequate to characterise a bladder mass. Spontaneous bladder rupture is notoriously difficult to diagnose, and in this series, only seven (23%) of patients were suspected of bladder rupture before surgery, with most cases being diagnosed at laparotomy. This is similar to a review by Reddy et al., where they reviewed cases of spontaneous bladder rupture without structural bladder abnormalities. They found that 36% of the 351 patients included in their review were diagnosed with spontaneous rupture of the bladder at the time of surgery.^2^ A low index of suspicion with nonspecific clinical signs are thought to be the contributing factor to this reduced pre‐surgical diagnosis.

In the broader population diagnosed with bladder cancer in developed countries, UCCs comprise about 90%, while SCC account for 5% of all tumours.^12^ In our study, a greater proportion of SCCs accounted for bladder rupture, significantly different from the reported incidence of UCC and SCC bladder tumours. This may be due to the more aggressive nature of bladder SCCs, which may cause more rapid invasion and destruction of the bladder wall integrity, although other factors could be considered, such as delayed presentation, reduced access to health care and advanced disease in certain geographical areas associated with high prevalence (e.g., schistosomiasis endemic regions).^12^ Patients with perforated bladders in the context of bladder cancer are likely to have invasive disease through the lamina propria and muscle.^14^ Bladder SCCs may grow more rapidly or have delayed presentation, have a greater propensity for invasion than UCCs and have a poorer prognosis.^12^

In our study, 30% of patients died during admission. Most patients recovered from the initial, acute phase, although a further 17% died within 6 months from subsequent cancer or complications. The other case reports did not report on mortality after discharge, likely due to a lack of follow‐up on these patients. Thus, almost half of the patients in this review died within 6 months of presentation, and this is likely an underestimate. In the study by Reddy et al. the mortality of patients who present with spontaneous bladder rupture due to non‐cancerous causes was 15%.^2^ Again, this reflects the aggressive nature of malignancies that lead to this presentation.

This is the first systematic review of bladder perforations in the context of bladder cancer, providing a descriptive analysis of the existing literature and characterising patients' epidemiology, presenting signs, management options, cancer histology and mortality. A limitation of this study is the small sample size of eligible cases and the heterogeneity of the cases and detail in the reports.

Spontaneous bladder perforation in the context of bladder cancer is a rare and easily overlooked cause of acute abdomen. The symptoms are nonspecific, investigations can be ambiguous and mortality is high. In patients presenting with peritonitic abdominal pain, clinicians should consider this diagnosis. A high suspicion should be alerted to this differential regarding additional preceding haematuria and urinary symptoms, especially in the absence of other pathologies during laparotomy. Patients have increased mortality with this diagnosis, highlighting the aggressive nature of this disease and raising important questions such as the role of emergency cystectomy, timing of systemic therapy and appropriate palliative care.

## AUTHOR CONTRIBUTIONS

*Review of articles for systematic review:* Johnson Da Huang, Emily Ximin Shao, and Handoo Rhee. *Manuscript generation:* Emily Ximin Shao and Johnson Da Huang. *Review of article:* Emily Ximin Shao, Johnson Da Huang, Chui Ming Tham, Eric Chung, and Handoo Rhee.

## CONFLICT OF INTEREST STATEMENT

None.

## Supporting information


**Table S1:** Characterisation of case reports of bladder cancer patients with spontaneous bladder rupture including gender, age, presenting signs and symptoms, histology and outcome.
