# Supplementary material for: Bladder malignancy as a cause of spontaneous bladder rupture: A systematic review
Source: BJUI Compass. 2023 Aug 30;5(1):12–6. doi: 10.1002/bco2.281 (PMC10764159; doi:10.1002/bco2.281)
Supplement: Supplementary file 1 — Table S1: Characterisation of case reports of bladder cancer patients with spontaneous bladder rupture including gender, age, presenting signs and symptoms, histology and outcome. [file BCO2-5-12-s001.docx]

Supplemental Table 1: Characterisation of case reports of bladder cancer patients with spontaneous bladder rupture including gender, age, presenting signs and symptoms, histology and outcome.

| Year | Author(s) | Sex | Age | Presenting signs and symptoms | Histology | Outcome |
| --- | --- | --- | --- | --- | --- | --- |
| 2023 | Huang et al. | M | 74 | Abdominal pain, chronic haematuria, fevers, night sweats, weight loss, vomiting, malaena, shortness of breath | UCC | Recovered, died due to metastatic disease |
| 2022 | Hagimoto et al.[1] | M | 86 | Abdominal pain | TCC | Discharged but died 74 days after initial diagnosis |
| 2022 | Sun et al.[2] | F | 57 | Abdominal pain and distension | Inflammmatory myofibrotic tumour | Unknown |
| 2021 | Badheeb et al.[3] | M | 74 | Abdominal tenderness and guarding | TCC | Recovered and discharged |
| 2021 | Saadi et al.[4] | M | 62 | Abdominal pain and deterioration of general condition | TCC | Recovered and then treated palliatively |
| 2020 | Asano et al.[5] | F | 52 | Impaired consciousness | SCC | Recovered, then died from cancer-related cachexia 10 weeks later |
| 2018 | Edwan et al.[6] | M | 56 | Diffuse abdominal pain, obstructive urinary symptoms | SCC | Recovered, died 6 months later of sepsis |
| 2014 | Oray et al.[7] | M | 56 | Abdominal pain, abdominal distension, left leg pain, difficulty walking, peritonism | Unspecified | Died during admission |
| 2014 | Hadjipavlou et al. [8] | M | 65 | Sudden onset lower abdominal pain, abdominal distension, bladder distension, urinary retention, peritonism | UCC | Full recover after 3 weeks |
| 2009 | Ohzawa et al. [9] | M | 69 | Lower abdominal pain, intermittent haematuria, peritonism | SCC | Died during admission after 2 months |
| 2009 | Ahmed et al. [10] | F | 47 | Generalised abdominal pain, peritonism | UCC | Slow recovery |
| 2007 | Rangarajan and Jayakar [11] | M | 30 | Urinary retention, dysuria | SCC | Recovered and discharged after 9 days |
| 2002 | Jayathillake et al. [12] | F | 72 | Sudden onset lower abdominal pain | SCC | Died during admission Day 10 |
| 2001 | Goel and Goel [13] | M | 55 | Peritonism | SCC | Recovered |
| 2000 | Chakravarti et al. [14] | M | 66 | Haematuria (minimally descriptive) | UCC | Recovered |
| 1998 | O'Neill and Alexander [15] | M | 46 | Sudden onset lower abdominal pain radiating to the left upper quadrant, intermittent haematuria, urinary frequency and urge incontinence, peritonism | UCC | Recovered and discharged after 2 weeks |
| 1998 | Atalay and Karamna [16] | F | 75 | Sudden onset abdominal pain, nausea, vomiting, peritonism | UCC | Died during admission Day 20 |
| 1994 | Rasmusen [17] | F | 61 | Sudden onset abdominal pain, peritonism | UCC | Died during admission 4 months |
| 1992 | Gough et al. [18] | F | 77 | Suprapubic pain radiating to the left iliac fossa, urinary incontinence, peritonism | SCC | Good recovery |
| 1991 | Sorensen et al. [19] | F | 51 | Recurrent UTIs, lower abdominal wall swelling and discolouration, peritonism | UCC | Died during admission day 3 |
| 1989 | Wujanto et al. [20] | F | 79 | Sudden lower abdominal pain | SCC | Died during admission day 24 |
| 1988 | Budd [21] | F | 79 | Generalised abdominal pain, urinary incontinence, dysuria, haematuria, peritonism | UCC | Recovered. Died later due to metastatic disease. |
| 1983 | Powell and Williams [22] | M | 42 | Lower abdominal pain, haematuria, urinary urgency, peritonism | UCC | Not specified |
| 1983 | Huffman et al. [23] | F | 49 | Abdominal pain, chronic urinary frequency, dysuria, abdominal tenderness | SCC | Recovered |
| 1981 | Jenkinson [24] | F | 73 | Lower abdominal pain, nausea, vomiting, anorexia, peritonism | SCC | Died during admission day 21. |
| 1976 | Agapitidis et al. [25] | M | 82 | Abdominal pain, vomiting, urinary retention, peritonism | Unspecified | Recovered and discharged day 20. |
| 1967 | Glashan [26] | F | 43 | Upper abdominal pain, vomiting, loose bowel motion, nocturia, generalised abdominal tenderness | UCC | Recovered slowly. |
| 1967 | Glashan [26] | M | 38 | Sudden onset generalised abdominal pain, peritonism | UCC | Not specified |
| 1959 | Bastable et al. [27] | M | 57 | Sudden onset lower abdominal pain, haematuria, peritonism | Unspecified | Died during admission day 4. |
| 1959 | Bastable et al. [27] | M | 57 | Sudden onset lower abdominal pain, peritonism | UCC | Recovered |

Male (M), female (F), urothelial cell carcinoma (UCC), squamous cell carcinoma (SCC).

1. Hagimoto H, Sano T, Kashima S, Yoshino T, Goto T, Sawada A, et al. Rapidly Progressive Bladder Cancer Diagnosed because of Spontaneous Bladder Rupture. Case Rep Urol. 2022;2022:4586199.

2. Sun Z, Qi L, Guo Z, Yuan W, Du Y, Gao H, et al. Case report: Emergency management of spontaneous rupture of the inflammatory myofibroblastic tumor of the urinary bladder. Frontiers in Oncology. 2022;12.

3. Badheeb A, Alkhanbashi O, Rakrouki S, Mahmood T, Alqannas M, Badheeb M, et al. Bladder rupture after pembrolizumab immunotherapy for bladder cancer: a case report. Pan Afr Med J. 2022;42:98.

4. Saadi MH, Mrad Dali K, Rahoui M, Sellami A, Ben Rhouma S, Nouira Y. Rupture of urinary bladder secondary to bladder carcinoma with extensive abdominal gangrene: A case report. Int J Surg Case Rep. 2021;81:105717.

5. Asano T, Ohtsuka Y. Spontaneous bladder rupture due to bladder carcinoma: A case report required emergency radical cystectomy. Urol Case Rep. 2020;33:101412.

6. Al Edwan GM, Mansi HH, Atta ONM, Shaban MM. Squamous cell carcinoma of the bladder presented with spontaneous intraperitoneal bladder rupture: A case report. Int J Surg Case Rep. 2018;48:61-4.

7. Oray D, Limon O, Ertan C, Ugurhan A. Spontaneous Bladder Rupture and Pelvic Fracture Due To Bladder Cancer. Turk J Emerg Med. 2014;14(3):139-41.

8. Hadjipavlou M, Tharakan T, Khan SA, Swinn M. Spontaneous bladder rupture of a urinary bladder with non-muscle invasive bladder cancer. BMJ Case Rep. 2014;2014.

9. H Ohzawa WA, Y Kondo, S Endo, T Morita, S Matsubara. . Urinary Bladder Rupture Associated With Squamous Cell Carcinoma Of The Bladder; A Cause Of Acute Peritonitis　The Internet Journal of Urology. 2009;7(2).

10. Ahmed J, Mallick IH, Ahmad SM. Rupture of urinary bladder: a case report and review of literature. Cases J. 2009;2:7004.

11. Rangarajan M, Jayakar SM. Rare case of spontaneous rupture of a urinary bladder carcinoma causing intestinal obstruction and peritonitis. Surgical Practice. 2007;11(1):44-7.

12. Jayathillake A, Robinson R, Al-Samarii A, Manoharan M. Spontaneous rupture of bladder presenting as peritonitis. N Z Med J. 2002;115(1164):U222.

13. Goel A, Goel A. Carcinoma urinary bladder presenting as acute abdomen. International Urology and Nephrology. 2001;33(3):491-2.

14. Chakravarti A, Day DW, MacDermott S. Extravesical transitional cell carcinoma as a result of implantation after perforation of the bladder. BJU Int. 2000;85(9):1150-1.

15. O'Neill GF, Alexander JH. Spontaneous bladder rupture in a patient with widespread superficial transitional cell carcinoma. Aust N Z J Surg. 1998;68(1):79.

16. Atalay AC, Karaman MI. Spontaneous rupture of a bladder with invasive bladder carcinoma. Int Urol Nephrol. 1998;30(6):723-4.

17. Rasmusen JS. Spontaneous bladder rupture in association with carcinoma. Scand J Urol Nephrol. 1994;28(3):323-6.

18. Gough M, McDermott EW, Lyons B, Hederman WP. Perforation of bladder carcinoma presenting as acute abdomen. Br J Urol. 1992;69(5):541-2.

19. Sørensen VT, Asklin B, Brunkwall P. Extraperitoneal perforation of the urinary bladder in association with urothelial cancer in a female. Scand J Urol Nephrol. 1991;25(4):277-8.

20. Wujanto R, Brough R, O'Reilly PH. Spontaneous perforation of squamous cell bladder carcinoma associated with hypercalcaemia. Br J Urol. 1989;63(6):647-8.

21. Budd JS. Spontaneous intraperitoneal rupture of the bladder in association with transitional cell carcinoma. Postgrad Med J. 1988;64(748):165-6.

22. Powell JM, Williams G. Spontaneous perforation of the bladder in association with malignant disease and schistosomiasis. Br J Urol. 1983;55(1):126.

23. Huffman JL, Schraut W, Bagley DH. Atraumatic perforation of bladder. Necessary differential in evaluation of acute condition of abdomen. Urology. 1983;22(1):30-5.

24. Jenkinson LR. Spontaneous intraperitoneal rupture of the urinary bladder. Postgrad Med J. 1981;57(666):269-70.

25. Agapitidis N, Papadopoulos N, Papastamatiou L. A rare case of spontaneous rupture of the urinary bladder. South Med J. 1976;69(2):244-5.

26. Glashan RW. Perforation as a complication of carcinoma of the bladder. Br J Urol. 1967;39(2):178-80.

27. Bastable JRG, Jode LRD, Warren RP. SPONTANEOUS RUPTURE OF THE BLADDER. British Journal of Urology. 1959;31(1):78-86.
